# Frontiers in *Shigella* Vaccine Development

**DOI:** 10.3390/vaccines10091536

**Published:** 2022-09-15

**Authors:** Calman Alexander MacLennan, Andrew Duncan Steele

**Affiliations:** Enteric & Diarrheal Diseases, Global Health, Bill & Melinda Gates Foundation, 500 5th Ave N, Seattle, WA 98109, USA

In recent years, there has been a resurgence of interest in the development of vaccines against *Shigella* driven by the growing awareness of the impact of this pathogen on global health. With multiple vaccines in clinical development, there has not been an easy way to access information on current lead candidates. Several vaccines have followed protracted development pathways involving multiple iterations of the same vaccine. Some iterations have been necessary to deal with excess reactogenicity or poor immunogenicity. Others have involved advancing a successful monovalent vaccine to a multivalent product with broad *Shigella* serotype coverage for global use. As a result, the development of many *Shigella* vaccines has been described piecemeal over several years and in multiple papers, none providing the full story of the vaccine they relate to.

For many of the current vaccines, up-to-date information has been difficult to obtain due to a hiatus between the completion of clinical trials and publication of trial findings. This gap can span several years during which time developers may present results at international meetings, though such opportunities have been few and far between during the recent COVID-19 pandemic. An interested reader may glean some information through ClinicalTrials.gov, though available information is usually quite limited.

This Special Issue of *Vaccines* serves to fill the current gap in available information on *Shigella* vaccines and provide such information in one place. The aim of the issue is to give the reader an up-to-date landscape of *Shigella* vaccines in clinical development with individual papers contributed by the vaccine developers themselves. We approached the developers of all *Shigella* vaccines in active clinical development known to us. From each developer we requested a contribution to the Special Issue, detailing the current status of their candidate vaccine and the development pathway followed to date.

*Shigella* is the main cause of diarrheal deaths in children over one year of age and the most common cause of bacterial diarrheal deaths globally. The most recent estimate from the Institute for Health Metrics and Evaluation (IHME) Global Burden of Disease (GBD) study is 148,202 deaths due to shigellosis in 2019, with 93,831 in children under 5-years of age. The attributable fraction of diarrheal deaths in GBD 2019 was 9.77% overall and 18.72% among under-5 s [1,2]. In contrast, the Maternal Child Epidemiology Estimation (MCEE) derived a lower value of 28,000 deaths among children under 5-years in 2013 [3] (Table 1).

A key difficulty with preventing shigellosis is the lack of a widely available diagnostic test. At present, a diagnosis of shigellosis is reliant on stool culture requiring specialized media which is not widely available in many *Shigella*-endemic settings. No rapid affordable point of care test exists and a diagnosis on clinical criteria alone is not possible [4]. Although shigellosis may present with dysentery, which has high specificity for *Shigella* infection, most cases present with watery diarrhea for which antibiotics are not routinely recommended by the WHO. A vaccine against *Shigella* could therefore have a major impact by preventing this important cause of diarrheal mortality.

Levels of antimicrobial resistance are increasing globally among *Shigella* isolates making shigellosis increasingly difficult to treat. Antimicrobial resistance among *Shigella* isolates is recognized as a major concern by both the WHO [5] and US Centers for Disease Control [6] which list *Shigella* as a critical pathogen for targeted intervention. The growing appreciation of vaccines as valuable tools for combatting antimicrobial resistance [7,8] adds to the need for a licensed vaccine against *Shigella*. *Shigella* have been on the WHO Product Development Vaccine Advisory Committee list of pathogens in urgent need of a vaccine for some time [9].

Children in low- and middle-income countries (LMICs) are disproportionately affected, making shigellosis a disease of poverty. Unfortunately, this has also served to make development of *Shigella* vaccines commercially unattractive and therefore of limited interest to the multinational vaccine manufacturers. Nevertheless, in recent years there has been an increase in charitable funding available for *Shigella* vaccine development from organizations including the Bill & Melinda Gates Foundation, Wellcome Trust, the US National Institutes of Health, and the European & Developing Countries Clinical Trials Partnership (EDCTP).

Shigellosis has well-recognized sequelae manifesting as linear growth stunting [10,11] and cognitive impairment [12] which result in further suffering and adverse economic impact. An effective vaccine against *Shigella* will save lives, increase economic growth and help curb global spread of antimicrobial resistance. It is clear that a *Shigella* vaccine will help address several of the Sustainable Development Goals including SDGs 1 (No Poverty), 3 (Global Health), 8 (Economic Growth), and 10 (Reduced Inequality) [13]. A *Shigella* vaccine is also attractive for adult travelers including military personnel serving in *Shigella*-endemic areas [14]. Hence, there is a potential dual market for a *Shigella* vaccine among travelers and the global pediatric population.

However, despite over a 100-years of development efforts, there has never been a widely licensed vaccine against shigellosis. There are several reasons for this, in addition to the lack of commercial incentive. Technically, it has proved difficult to develop an effective vaccine with many candidates proving to be either too reactogenic or insufficiently immunogenic to be efficacious. As a consequence, *Shigella* has become a graveyard of vaccine development. Since there are over 50 serotypes of *Shigella*, a multivalent vaccine approach is likely to be required for a sufficiently effective vaccine, though most candidates to date have been in monovalent format. From the Global Enteric Multicenter Study (GEMS) it has been estimated that a 4-valent vaccine including *S. flexneri* 2a, 3a, 6 and *S. sonnei* would cover up to 75% of global strains and up to 93% through cross-protective epitopes [15].

We were pleased to receive contributions from six of the eight vaccine developers we approached. Most of the vaccines are predicated on *Shigella* O-antigen and induce serum and mucosal antibodies to this molecule. A variety of platform technologies are being utilized to engineer these vaccines including bioconjugation, synthetic chemistry, outer membrane vesicles, physical mixtures of antigens and whole cell/live attenuated bacteria.

Two of the papers report the hitherto unpublished findings of recent clinical trials. Mo and colleagues from Beijing Zhifei Lvzhu Biopharmaceuticals describe the Phase 2 study of their bivalent *S. sonnei/S. flexneri* 2a O-antigen conjugate vaccine [16]. This is now being assessed in a Phase 3 study in China and is the most advanced *Shigella* vaccine and, as far as we are aware, the only multivalent *Shigella* vaccine to be assessed in a Phase 3 study to date [17]. The findings from this study will provide valuable information on the likelihood of other second-generation O-antigen-based *Shigella* subunit vaccines being effective in the target population of young children.

Adopting a very different approach with a live attenuated *Shigella* vaccine, Girardi and colleagues from Eveliqure, Vienna, Austria, present findings from a Phase 1 study with their candidate vaccine ShigETEC [18]. This vaccine has also progressed to Phase 2 studies, and an early readout of efficacy will be obtained from a controlled human infection model (CHIM) study. The vaccine is particularly intriguing as it lacks both O-antigen and Ipa proteins, the best characterized targets of protective immunity against *Shigella*, so must rely on immune responses to less well-known antigens to confer protection.

Contributions from other vaccine developers serve as valuable and comprehensive up-to-date reviews of four promising subunit candidates. Martin and Alaimo describe the development of the LimmaTech bioconjugate *Shigella* vaccine program [19]. Bioconjugation, where glycoconjugate vaccine is produced by genetically engineered *E. coli*, is a relatively new vaccine technology. The current multivalent iteration of the vaccine is completing a Phase 2 study in Kenyan children [20] and is the most advanced 4-valent *Shigella* vaccine.

Micoli and colleagues describe another multivalent *Shigella* vaccine approach using a different novel platform technology which is about to be tested in Kenyan children [21]. GMMA (Generalized Modules for Membrane Antigens) technology is essentially a native outer membrane vesicle approach predicated on blebs of outer membrane shed from Gram-negative bacterial such as *Shigella*. This is thought to have major advantages in relation to manufacturability and affordability. Though the vesicles are regarded as a delivery vehicle for O-antigen, they also contain a wide array of other outer membrane antigens, particularly proteins, that can contribute to protective immunity.

A third conjugate vaccine approach is described by Phalipon and Mulard from the Pasteur Institute [22]. They have taken a synthetic approach to the production of well-defined short chain O-antigens which are conjugated to tetanus toxoid. Though data are only so far available from a phase 1 study with a monovalent *Shigella flexneri* 2a vaccine, this candidate is also being tested in Kenyan children and in a CHIM study, while at the same time a multivalent version of the vaccine is being developed.

The final vaccine contribution to the Special Issue is from Turbyfill and colleagues from the Walter Reed Army Institute for Research (WRAIR) [23]. Their ‘Invasin Complex’ approach is unique among the *Shigella* subunit vaccines, consisting of a physical mixture of *Shigella* lipopolysaccharide and Ipa proteins with no conjugation. The long history of this vaccine through several iterations, accompanied by a change from oral to parenteral administration, is carefully described.

With one *Shigella* vaccine candidate already in a Phase 3 study and other promising candidates advancing towards Phase 3, Pavlinac and colleagues cover key considerations for Phase 3 *Shigella* trial design [24]. Such work, including decisions on clinical trial endpoints, trial size and location, is key for maximizing the prospect of successful Phase 3 trials.

To those working in the field of enteric vaccines, development of a vaccine against the main bacterial cause of diarrheal deaths is obvious. However, in their contribution, Hausdorff and colleagues point out the challenges a licensed *Shigella* vaccine may face on the pathway to introduction [25]. Although a global health priority for many years, a *Shigella* vaccine will have stiff competition at the national level from other vaccine priorities. In their commentary, Hausdorff et al. lay out the key components that will be required for a full *Shigella* vaccine value proposition, including impact on linear growth faltering and cognitive impairment mentioned above.

A historical perspective on the development of *Shigella* vaccines is provided by Herrera and colleagues from the University of Nevada [26]. It is sobering to appreciate that attempts to develop *Shigella* vaccines have been ongoing for 120 years with no license vaccine available. This perhaps underlies the difficulties faced by vaccine developers over the years with no easy wins achieved by either the whole cell inactivated approaches of the first half of the 20th century or the live attenuated approaches adopted in the second half of the 20th century and early years of the 21st century.

Further historical perspective is provided by Cohen and colleagues from Tel Aviv University who describe the breakthrough achieved in *Shigella* vaccinology by a *Shigella sonnei* O-antigen conjugate vaccine developed at the US National Institutes of Health [27]. This work has proved pivotal as it provided vital proof-of concept for the parenteral subunit glycoconjugate vaccine approach with efficacy demonstrated in young Israeli soldiers 25 years ago [28] and subsequently in Israeli children down to three years of age [29]. The work was also critical for establishing serum O-antigen IgG as a correlate of protection against shigellosis [30].

Finally, we provide, in a separate article, a description of the *Shigella* vaccine pipeline putting the various current candidates covered in this Special Issue in context with vaccines whose development has been halted for one or other reason [31]. This last article in the Special Issue serves to group candidates by broad technology approach, vaccine developer and serotype coverage. Although it is exciting to see the promising group of current candidate vaccines, it is salutary to appreciate how many have failed over the years.

In summary, this Special Issue presents a current snap-shot of the state of *Shigella* vaccine clinical development in the historical context of many years of unsuccessful attempts to develop such a vaccine. Though *Shigella* vaccine development has proved a difficult path, there is much to be hopeful about with a range of promising technological platforms being applied to this challenge and several candidates already in the target population of young children in LMICs. In the next few years, we will find out which are successful.

## Figures and Tables

**Table 1:** Estimated deaths and pathogen attributable fraction of diarrheal deaths due to *Shigella* for all ages and for children under-5 years.

Source	Deaths		Pathogen Attributable Fraction (PAF)	
	Under-5 Years	All-Age	Under-5 Years	All-Age
IHME ^1^* Global Burden of Disease 2019 [1,2]	93,831 (35,860–185,931)	148,202 (61,975–284,541)	18.72% (7.86–34.55)	9.77% (4.24–18.42)
MCEE ^2^ 2013 [3]	28,000 (12,000–53,000)			

^1^ IHME Institute for Health Metrics Evaluation, ^2^ MCEE Maternal Child Epidemiology Estimation (formerly the Child Health Epidemiology Reference Group, or CHERG). * Source: Institute for Health Metrics and Evaluation. Used with permission. All rights reserved.
